# Do boys with MAOA_LPR*2R allele present cognitive and learning impairments?

**DOI:** 10.1590/1980-5764-DN-2021-0071

**Published:** 2022-05-13

**Authors:** Emanuelle de Oliveira Silva, André Henrique Barbosa de Carvalho, Giulia Moreira Paiva, Carolina Andrade Jorge, Gabriella Koltermann, Jerusa Fumagalli de Salles, Vitor Geraldi Haase, Maria Raquel Santos Carvalho

**Affiliations:** 1 Universidade Federal de Minas Gerais, Instituto de Ciências Biológicas, Programa de Pós-graduação em Neurociências, Belo Horizonte MG, Brazil.; 2 Universidade Federal de Minas Gerais, Ecologia e Evolução, Instituto de Ciências Biológicas, Programa de Pós-Graduação em Genética, Departamento de Genética, Belo Horizonte MG, Brazil.; 3 Universidade Federal do Rio Grande do Sul, Programa de Pós-Graduação em Psicologia, Porto Alegre RS, Brazil.; 4 Universidade Federal de Minas Gerais, Departamento de Psicologia, Faculdade de Filosofia e Ciências Humanas, Belo Horizonte MG, Brazil.; 5 Universidade Federal de Minas Gerais, Faculdade de Filosofia e Ciências Humanas,Programa de Pós-Graduação em Psicologia: Cognição e Comportamento, Departamento de Psicologia, Belo Horizonte MG, Brazil.; 6 Instituto Nacional de Ciência e Tecnologia sobre Cognição, Comportamento e Ensino, São Carlos SP, Brazil.; 7 Universidade Federal de Minas Gerais, Instituto de Ciências Biológicas, Departamento de Genética, Ecologia e Evolução, Belo Horizonte MG, Brazil.

**Keywords:** Monoamine Oxidase, Working Memory, Intelligence, Learning Disabilities, Dyscalculia, Neuropsychology, Monoaminoxidase, Memória de Trabalho, Inteligência, Deficiências da Aprendizagem, Discalculia, Neuropsicologia

## Abstract

Monoamine oxidase A (*MAOA*) polymorphisms have been associated with antisocial disorders. Less attention has been paid to the cognitive functioning of individuals with different *MAOA* alleles. No study has described the cognitive phenotype associated with the less frequent, low enzyme activity allele, MAOA_LPR*2R.

**Objective:**

We describe the cognitive correlates of boys having MAOA_LPR*2R allele, ascertained in a sample of school children with normal intelligence, not referred for behavioral disorders.

**Methods:**

Participants were eight boys, attending from the second to fifth grades in state-run schools. They were identified among 712 children with typical general cognitive ability, genotyped for MAOA_LPR polymorphism. Participants were assessed with general intelligence, mathematics and spelling achievement, and verbal and visuospatial working memory tests. Neuropsychological performance was compared to published standards, using 1 SD below the mean as a cutoff value for low performance.

**Results:**

Intelligence of boys with MAOA_LPR*2R allele varied from above average (N=2) to low average in the other children. Five out of eight boys with the MAOA_LPR*2R allele had low mathematics achievement, and three presented additional difficulties with spelling. Four out of eight children had low short-term and working memory performance.

**Discussion:**

This is the first study describing cognitive correlates and school performance in boys having the MAOA_LPR*2R allele. Having this allele, and therefore, probably low MAO-A activity, does not necessarily imply low intelligence or low school performance. However, learning difficulties, particularly in math, and low working memory performance were observed in boys having this allele. This suggests a role of *MAOA* in learning difficulties.

## RESUMO.

Polimorfismos da monoaminoxidase A (MAOA) são associados a transtornos antissociais. Menos atenção tem sido dada ao funcionamento cognitivo de indivíduos com diferentes alelos de MAOA. Nenhum estudo descreveu o fenótipo cognitivo associado ao alelo menos frequente, de baixa atividade enzimática, MAOA_LPR*2R.

**Objetivo:**

Descrevemos os correlatos cognitivos de meninos com o alelo MAOA_LPR*2R, identificados em uma amostra de escolares com inteligência normal, não encaminhados por transtornos de comportamento.

**Métodos:**

Oito meninos com o alelo MAOA_LPR*2R foram identificados entre 712 crianças genotipadas, com inteligência típica, que cursavam do 2º ao 5º ano em escolas públicas. Foram avaliados: inteligência, desempenho em matemática e ortografia, memória de trabalho verbal e visuoespacial. O desempenho foi comparado a normas publicadas, utilizando-se 1 desvio padrão (DP) abaixo da média como ponto de corte para desempenho rebaixado.

**Resultados:**

A inteligência dos meninos com alelo MAOA_LPR*2R variou de acima da média (N=2) a médio-inferior nas demais crianças. Cinco dos oito meninos com alelo MAOA_LPR*2R apresentaram desempenho rebaixado em matemática e três apresentaram dificuldades adicionais em ortografia. Quatro dos oito meninos apresentaram baixo desempenho de memória de curto prazo e de trabalho.

**Discussão:**

Este é o primeiro estudo a descrever os correlatos cognitivos e o desempenho escolar em meninos com alelo MAOA_LPR*2R. Ter esse alelo não significa necessariamente baixa inteligência ou baixo desempenho escolar. No entanto, dificuldades de aprendizagem, principalmente em matemática, e desempenho rebaixado da memória de trabalho foram observados em mais da metade dos meninos com esse alelo. Isso sugere um papel do MAOA nas dificuldades de aprendizagem.

## INTRODUCTION

Monoamine oxidase A (MAO-A) is a mitochondrial enzyme involved in several different metabolic pathways, processing endogenous as well as exogenous metabolites^1^. MAO-A is one of the enzymes that catalyzes neurotransmitters in the central nervous system, including dopamine, serotonin, and norepinephrine^2^. MAO-A contributes to the regulation of the half-life of these neurotransmitters and, consequently, contributes to the regulation of their availability in the synaptic cleft. The *MAOA* gene is ubiquitously expressed, but its behavioral effects vary according to the brain region and the expression levels of other partners in the catecholamine availability regulation, such as catechol-*O*-methyltransferase (COMT) or MAO-B^1^.

The first clearcut evidence for an MAO-A contribution for behavioral phenotypes emerged, when a pathogenic variant in the *MAOA* gene was identified in 14 individuals of a Dutch family^3^. Men in this family presented nonsyndromic, mild intellectual disability or borderline intelligence. Eight of the affected men in this family were examined. All presented stereotyped hand movements described as “wringing, plucking, and fiddling.” In addition, their behavior was characterized by shyness, aggressive outbursts, stabbing, and fighting. Arson was reported for two of them. They also presented abnormal sexual behavior, characterized by exhibitionism, voyeurism, grasping, or holding of female relatives, and rape or rape attempts^4^. Females in this family were reportedly unaffected. Subsequently, evidence for the role of MAO-A in aggression and antisocial behaviors was found in many studies^5^ ^,^ ^6^ ^,^ ^7^ ^,^ ^8^.

Polymorphisms in *MAOA* have been associated with several neuropsychiatric disorders such as mood disorders and attention deficit and hyperactivity disorder (ADHD)^9^ ^,^ ^10^ ^,^ ^11^. Additionally, *MAOA* polymorphisms have been associated with autism^7^ ^,^ ^12^. The most investigated polymorphism is a variable number of tandem repeats (VNTR) located in the *MAOA* gene promoter region and identified as MAOA_LPR (also known as MAOA-uVNTR). The monomer of the repetitive element is 30 bp long. MAOA_LPR has two frequent alleles (i.e., MAOA_LPR*3R and MAOA_LPR*4R) and three less common ones (i.e., MAOA_LPR*2R, MAOA_LPR*3.5R, and MAOA_LPR*5R). This is a regulatory polymorphism. The MAOA_LPR*3.5R and MAOA_LPR*4R alleles have been associated with a higher gene transcription and enzyme activity and are referred to as MAOA-H. Meanwhile, MAOA_LPR*3R and MAOA_LPR*5R present low enzymatic activity and are referred to as MAOA-L alleles^13^. It has been described that the MAOA_LPR*2R allele presents 25-30% of the transcriptional activity when compared to the MAOA_LPR*4R allele^6^. Consequently, MAOA_LPR*2R would also be a low activity allele. A synthesis of the studies describing MAOA_LPR*2R effects is presented in Table 1. *MAOA* polymorphisms have been consistently associated with disruptive and antisocial behavioral disorders. Most studies have focused on the behavioral aspects, with considerably less studies investigating cognitive aspects. Less attention has also been given to the less common alleles such as MAOA_LPR*2R. In this study, the cognitive correlates of the MAOA_LPR*2R allele are investigated.

**Table 1.** Studies investigating the association between MAOA*2R and behavior disorders (PubMed search using MAOA, MAOA AND working memory, MAOA 2R, MAOA AND intelligence, MAOA 2-repeat allele).

References	n	Sex	Population/cohort	Phenotypes	Findings and conclusions
Guo et al.^6^	2,524	Both sexes	National Longitudinal Study of Adolescent Health (Add Health)	Delinquent behavior	Boys with the MAOA_LPR*2R allele had twice the chance of presenting delinquent and violent behaviors when compared with participants with other alleles. The same effect is observed in girls but with less intensity.
Åslund et al.^19^	1,825	Both sexes	Survey of Adolescent Life in Vestmanland 2006 (SALVe-2006)	Delinquent behavior	MAOA_LPR genotype (one short variant for boys and one or two long variants for girls) showed a significant effect on delinquency when controlled for maltreatment.
Roettger et al.^22^	6,001	Males	National Longitudinal Study of Adolescent Health (Add Health)	Delinquent behavior	The relationship between delinquency and the MAOA_LPR*2R allele decreases in participants who were close to their biological or adoptive father, but not in those close to their mother.
Beaver et al.^23^	2,574	Males	National Longitudinal Study of Adolescent Health (Add Health)	Violent behaviors	African Americans carrying the MAOA_LPR*2R allele were more likely to engage in violent behaviors such as shooting or stabbing someone when compared to other MAOA_LPR genotypes.
Beaver et al.^24^	167/174	Males	National Longitudinal Study of Adolescent Health (Add Health)	Anti-social phenotypes	African Americans with MAOA_LPR*2R allele had higher scores on an antisocial phenotype scale. Individuals with the MAOA_LPR*2R allele were also significantly more likely to be arrested, when compared to individuals with other alleles. There were no data for Caucasians.
Daw and Guo^25^	2,167	Both sexes	National Longitudinal Study of Adolescent Health (Add Health)	Contraceptive use	Females carrying the MAOA_LPR*2R allele have higher odds of having unprotected sex. The authors did not find this association in males.
Stetler et al.^26^	89	Male	Imprisoned population	Violent behaviors	Violent crime charges were significantly more frequent in carriers of MAOA_LPR*2R or MAOA_LPR*3R alleles.
Barnett et al.^27^	6,000	Both sexes	Avon Longitudinal Study of Parents and Children (ALSPAC)	Cognitive function	MAOA_LPR alone did not show a significant effect on cognitive function. The authors found an association between MAOA_LPR and COMT Val158Met genotypes with better working memory.
Belsky and Beaver^28^	1,586	Both sexes	National Longitudinal Study of Adolescent Health (Add Health)	Self-regulation and adolescence parenting	Under different environmental conditions, MAOA_LPR could be one moderator of parenting and self-regulation in boys.
Rommelse et al.^29^	545	Both sexes	Dutch part of the International Multicenter ADHD Genetics (IMAGE) cohort	ADHD and neuropsychological functioning	One of the haplotypes was associated with poorer motor control in boys and with better visuospatial working memory in girls.
Chien et al.^30^	1,074	Male	In custody population	Heroin dependence	MAOA_LPR polymorphism does not appear to be involved in heroin dependence.
Ko et al.^31^	50	Males	ADHD and non-ADHD population	ADHD	ADHD carriers of rs1137070 T allele had higher activation of pars opercularis when compared with carriers of C allele.

Epigenetic studies comparing the degree of methylation of *MAOA* gene promoter between participants presenting antisocial behavior and normal controls also provided evidence that low *MAOA* activity (aka, high promoter methylation levels) is associated with antisocial personality^14^ ^,^ ^15^. A complex interaction with alleles in other genes has been described. For example, low activity alleles in both *MAOA* and *COMT* have been associated with higher adrenocorticotropic responses and cortical levels^16^ ^,^ ^17^. Stressful interactions in early life are a risk factor for antisocial disorders in male individuals with low *MAOA* activity, but not in females^5^. Such gene-gene and gene-environment effects help understanding the individual variation in the responses to stressful life experiences. For example, gene-environment interaction involving low activity alleles in *COMT* and *MAOA* genes and academic pressure has been reported^18^. *MAOA* maps to the short arm of the X-chromosome and, therefore, males are hemizygous, while females are homozygous or heterozygous. Consequently, allele/genotype effects vary depending according to sex. For example, the presence of low activity *MAOA* alleles in males has been associated with higher susceptibility to environmental stressors. On the contrary, higher sensitivity to environmental stressful influences has been associated with the presence of high activity *MAOA* alleles in females^19^ ^,^ ^20^ ^,^ ^21^.

Considering MAO-A relevance in the regulation of the half-life of neurotransmitters such as serotonin, norepinephrine, and dopamine, a large number of studies have been conducted, investigating the association of *MAOA* genotypes and different behavioral traits. Due to their higher frequencies, most studies consider only the MAOA_LPR*3R and MAOA_LPR*4R genotypes; sometimes, MAOA_LPR*3.5R is added to the MAOA_LPR*4R genotype. In comparison, the effects of MAOA_LPR*2R are less understood and investigated. Most known effects are related to antisocial behaviors and delinquency. These effects are usually associated with specific environmental conditions that can increase or decrease the genotype effects. Closeness to a father could moderate the MAOA_LPR*2R effect for delinquency over time^22^. Effects of maltreatment and other adverse conditions may also be mediated by MAOA_LPR*2R. Boys, who underwent maltreatment in childhood and who were hemizygous for the MAOA_LPR*2R allele, were more likely to commit infractions in adolescents or adulthood^19^.

Although several studies have shown the relationship between MAOA_LPR*2R and antisocial behaviors when associated with other environmental conditions, there is evidence for this effect without any apparent environmental influence. In a sample composed of Caucasian and African American men, the group carrying the MAOA_LPR*2R allele had higher chances of stabbing or shooting someone at least once in life, when compared with individuals having any other *MAOA* allele^23^. As MAOA*2R presents a higher frequency among African Americans and to correct for the economical risk factors associated, skin color was included in the analytic models. A possible source of bias in such studies is the low frequency of the MAOA_LPR*2R allele in Caucasian populations^24^, and large samples are required for the investigation of MAOA_LPR*2R effects in such populations. In addition to antisocial behavior, the MAOA*2R allele is also associated with impulsive behaviors. It has been shown that females carrying this allele had higher odds of having unprotected sex^25^.

Most studies on the effects of MAOA_LPR*2R allele were conducted in samples of individuals presenting antisocial behaviors^8^ ^,^ ^19^ ^,^ ^22^ ^,^ ^24^ ^,^ ^25^ ^,^ ^26^. There are no studies investigating the neuropsychological profile of children from the general population. In the present study, we review the literature and describe the neuropsychological characteristics observed in school boys having the MAOA_LPR*2R genotype. PubMed searches were done using the terms MAOA, MAOA AND working memory, MAOA 2R, MAOA AND intelligence, and MAOA 2-repeat allele. The search results are presented in Table 1.

In general, cognitive-behavioral characteristics associated with the MAOA_LPR*2R have been less explored than those with the more frequent phenotypes (Table 1). With the exception of one study^27^ ^,^ ^28^ ^,^ ^29^, the literature on phenotypes associated with the MAOA_LPR*2R genotype has explored the maladaptive behavioral more than the cognitive traits. In this study, we assessed cognitive abilities (i.e., intelligence, working memory, and numerical-arithmetic abilities) of eight male school children with the MAOA_LPR*2R genotype, who had normal intelligence and who were identified from a larger population sample. The hypothesis explored is that individuals with the MAOA_LPR*2R genotype may present difficulties with school achievement and working memory.

## METHODS

### Ethics in research

The selected participants are from two research projects with population data. Both projects were submitted and approved by the local ethics research board and complied with the Helsinki research principles for human beings (Project 1: “Developmental dyscalculia in school-age children: population screening and characterization of cognitive and genetic-molecular aspects” - COEP-UFMG: ETIC 42/08; Project 2: “Endophenotypes of learning difficulties in mathematics” - COEP-UFMG: CAAE 15070013.1.0000.5149). All children were authorized by their parents, through a written informed consent. Children’s participation was also conditioned to their oral consent. The evaluation was conducted in a separate quiet room that had been arranged by the school.

### Participants

Data were obtained from two research projects in which 712 children with intelligence above the 15th percentile (PR), aged between 7 and 11 years, were neuropsychologically assessed at their state-run schools and genotyped for the MAOA_LPR polymorphism. Children were attending state-run schools. Eight boys were hemizygotes for MAOA_LPR*2R. The children having the MAOA_LPR*2R allele attended the second (n=3), third (n=3), and fifth (n=2) grades. Neuropsychological data from the boys with MAOA_LPR*2R were compared with those of the published test norms (Table 2).

**Table 2.** Neuropsychological instruments.

Construct	Instrument	References
General cognitive abilities (Intelligence)	Raven’s Coloured Progressive Matrices (CPM)	Angelini et al.^32^
School achievement	TDE - Arithmetic subtest and Spelling subtest	Oliveira-Ferreira et al.^33^, and Gomides et al.^34^
Verbal and visuospatial short-term and working memory	WISC-III Digits (Verbal Short Term and Working Memory) and Corsi Blocks	Figueiredo and Nascimento^35^, and Galera and Souza^36^
Numerical and arithmetic abilities	Arabic number dictation, Addition, Subtraction, Multiplication	Gomides et al.^34^

WISC-III: Wechsler Intelligence Scale for Children III. With exception of Raven’s CPM, normative data were obtained from reference 33.

### Assessment

Children were assessed at their schools. The instruments were applied by specially trained psychology undergraduate and graduate students. The Raven’s Coloured Progressive Matrices (CPM), Arabic Number Dictation, and TDE Spelling subtest were applied in groups of an average of six children. The TDE - Arithmetic subtest; WISC-III Digits and Corsi Blocks; and Addition, Subtraction, and Multiplication tasks were applied in individual sessions. The description and references of the instruments used in the neuropsychological assessment are presented in Table 2.

### Genotyping

Genomic DNA extraction was conducted using a proteinase K/salting out adapted method^37^ ^,^ ^38^. The protocol is available under request. DNA quantity and purity were assessed using spectrophotometry in a Nanodrop spectrophotometer. The MAOA_LPR was genotyped by fragment analysis in a capillary electrophoresis sequencer. PCR primer sequences were obtained from the literature^39^: MAOA-LPR forward: 5’-FAM CCCAGGCTGCTCCAGAAACATG-3’ and MAOA-LPR reverse: 5’-GTTCGGGACCTGGGCAGTTGT-3’. The PCR consisted of 50 ng of total DNA, 10 pmol of each primer, 2 μg of Taq DNA polymerase, 5 μL of 5× buffer (Phoneutria Biotechnology, Belo Horizonte, Brazil), 2 μL of DMSO 100%, 20 mM dNTP, and Milli-Q water to a total volume of 25 μL. PCR cycling was composed of a 5 min initial denaturation step at 94°C, followed by 25 cycles of 94°C for 30 s, 56°C for 20 s, and 72°C for 30 s, and the last extension step of 5 min at 72°C. Amplicons were analyzed in an ABI 3730 capillary sequencer (Thermo Fisher Scientific), using the GeneScan™ 1200 LIZ^®^ Size Standard. Genotypes were obtained in the Applied Biosystems^®^ Sizing Analysis module Peak Scanner Software, version 3.0, available online in the Thermo Fisher Cloud. According to the fragment sizes, alleles were classified as 2-repeat (MAOA*2R), 183 bp; 3-repeat (MAOA*3R), 213 bp; 3.5-repeat (MAOA*3.5R), 229 bp; 4-repeat (MAOA*4R), 243 bp; and 5-repeat (MAOA*5R), 373 bp.

### Statistical analysis

**Figure 1.**
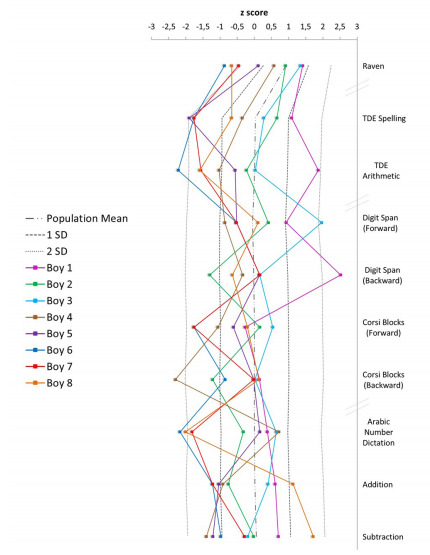
Quantitative results of the neuropsychological assessment for each boy having the MAOA_LPR*2R allele.

Data were qualitatively analyzed, comparing z-scores in the neuropsychological tests for the MAOA*2R individuals with those of the published norms (Table 2) (40). The z-scores for Raven’s CPM, Digit span, and Corsi Blocks were standardized by age. z-scores for TDE, simple addition, subtraction and multiplication, and transcoding were standardized by grade (Figure 1). Scores lower than 1 SD below the mean were characterized as low performance.

## RESULTS

### Neuropsychological assessment results

Allelic and genotype frequencies are shown in Supplementary Table 1. The qualitative results of the neuropsychological assessments are presented in Table 3. Quantitative neuropsychological results for children with MAOA_LPR*2R are presented in Figure 1 and Supplementary Table 2, and their results are shown in Supplementary Figure S1. The neuropsychological performance among boys with the MAOA_LPR*2R genotype was variable, with a completely normal neuropsychological examination in two out of eight boys. The most frequent findings were abnormally low performance in verbal and/or visuospatial working memory (VSWM) (4/8), math learning difficulty (5/8), and spelling difficulties (3/8). Low performance in working memory and school achievement tests become salient when data for all children with MAOA_LPR*2R are compared in the same figure (Figure 1 and Supplementary Figure S1).

**Table 3.** Qualitative neuropsychological results for the boys having the MAOA_LPR*2R allele.

Initials	Grade/age	Neuropsychological results
Boy 1	5th grade/10 years	Superior normal intelligence (1.4 SD above mean). Verbal working memory (2.5 SD above mean) and arithmetic achievement superior (1.8 SD above mean) for age. Typical for age performance in visuospatial short and working memory tasks; spelling achievement.
Boy 2	3rd grade/8 years	Normal intelligence. Typical for age performance in verbal and visuospatial short-term memory; arithmetic; and spelling achievement. Low performance on visuospatial and verbal working memory tasks.
Boy 3	3rd grade/7 years	Superior normal intelligence (1.3 SD above mean). Typical for age performance in verbal and visuospatial short-term and working memory; arithmetic; and spelling achievement.
Boy 4	3rd grade/ 9 years old	Normal intelligence. Typical for age performance on verbal short-term and working memory, and spelling achievement. Low performance in visuospatial short-term and working memory, and arithmetic achievement.
Boy 5	2nd grade/7 years	Normal intelligence. Typical for age performance in verbal and visuospatial short-term and working memory; and in spelling tasks. Low achievement in spelling and arithmetic tasks.
Boy 6	2nd grade/7 years	Normal intelligence. Typical for age performance in verbal and visuospatial short memory; visuospatial working memory. Low performance in a visuospatial short-term memory task, arithmetic, and spelling achievement.
Boy 7	2nd grade/7 years	Normal intelligence. Typical for age performance in verbal and visuospatial working memory, verbal working memory. Low performance in visuospatial short memory, spelling, and arithmetic achievement.
Boy 8	5th grade/11 years	Normal intelligence. Typical for age performance in verbal and visuospatial short-term and working memory, and spelling achievement. Low achievement in arithmetic.

## DISCUSSION

The aim of this study was to describe the results of a neuropsychological assessment of eight healthy, school-age boys with MAOA_LPR*2R genotype. A variable neuropsychological profile was observed in boys with normal intelligence. One of the children evaluated had a typical performance for his age in all tasks. ­However, a tendency toward difficulties in visuospatial and verbal short-term and working memory was observed. Furthermore, most children had difficulties in school performance, especially in arithmetic. A smaller number presented difficulties in spelling. In the following sections, results related to intelligence, working memory, and school achievement are discussed.

General intelligence in the participants was normal, compared to the population standards. This was expected as an intelligence above the PR15 was used as an inclusion criterion. Two boys with MAOA_LPR*2R genotype scored 1.3 SD above the intelligence mean. The intelligence of the other children was situated in the low normal range. Intelligence is a neuropsychological function with high heritability^41^. An association between *MAOA* and IQ has been found in several studies^42^, usually in connection with behavioral disorders. A study evaluated the predictive effect of *MAOA* on the intelligence of children with ADHD, in which approximately 40% of the sample had comorbidities with conduct disorder and oppositional defiant disorder. The results indicated that MAOA predicts the IQ of these children, while COMT Val158Met independently does not. An interaction was also found between COMT ­*Val158Met* genes and MAOA_LPR*2R and MAOA_LPR*4R polymorphisms with effect only on the performance IQ of the children in the sample^43^. Healthy women with the homozygous MAOA_LPR*4R allele had a higher intelligence than those who carry the homozygous MAOA_LPR*3R allele^42^.

Four of eight children were impaired in working memory tasks. Impairments in VSWM were more frequent than impairments in verbal working memory. Working memory deficits have been observed in individuals with disruptive behavioral disorders^44^ ^,^ ^45^ ^,^ ^46^. The literature suggests associations between *MAOA* polymorphisms and both verbal and VSWM. An association between verbal working memory mechanisms and the activation levels in *pars opercularis* was observed in adult individuals with ADHD. These individuals exhibited higher levels in the *pars opercularis* compared to controls when performing an N-back task. An SNP in *MAOA* (rs1137070) was described as a moderator of *pars opercularis* activation in these individuals. The effect of *MAOA* on *pars opercularis* activation was only significant in the carriers of the rs1137070 T/T genotype^22^. ­Evidence also indicates that MAOA is implicated in the neurobiological regulation of VSWM activity. VSWM deficits are a correlate of maladaptive behaviors^47^. Carriers of the allele A for the SNP rs6609257 exhibited higher cortical activity in the frontal, parietal, and occipital regions associated with working memory^47^. Alleles associated with higher *MAOA* activity modulate responses of the ventrolateral prefrontal cortex to VSWM tasks^48^.

Of the eight participants, five performed below 1 SD on the math achievement test. To the best of our knowledge, no previous investigation has addressed the impact of MAOA_LPR polymorphisms on school achievement. Disruptive behavioral disorders are not considered learning disorders. However, disruptive behavioral disorders are consistently associated with low achievement and school dropout^49^. As low school performance, especially in mathematics, was detected in individuals with MAOA_LPR*2R, it is possible to suggest that polymorphisms in MAOA_LPR*2R may also be associated with school underachievement in children with normal intelligence. A search on PubMed using “math achievement OR mathematics AND MAOA” in March 2021 yielded no results. This could be a venue for new research, as the results presented here suggest an association between the MAOA_LPR*2R allele and math achievement. The association between MAOA polymorphisms and mathematics achievement may be mediated by working memory mechanisms and could, eventually, be also moderated by COMT Val158Met polymorphisms^50^.

This study has some limitations. It is an exploratory study with a small sample size and we did not assess behavioral constructs. However, this is the first study investigating the cognitive correlates of a relatively infrequent *MAOA*-related genotype with potential neuropsychiatric and educational implications. This study fills in the gap of information on the effects of the MAOA_LPR*2R allele in children not selected due to antisocial behavior or intellectual disability.

An interesting finding in the present study is the phenotypic variability observed among the boys having the MAOA_LPR*2R allele, ranging from high performance in all tests to low performance in working memory tasks and learning difficulties associated with low but normal intelligence. This finding suggests that the effects of MAOA_LPR*2R are modulated by a multifactorial context, which includes possible environmental as well as genetic background effects. The findings reported here reinforce the concept that genetic polymorphisms affect behavior and school achievement in children with normal intelligence. At the population level, school achievement is a multifactorial characteristic. Although our results suggest an association between this polymorphism and school achievement, it is necessary to be parsimonious regarding its effects. The genetic architecture of behavioral traits is highly polygenic; MAOA_LPR*2R polymorphism is one risk factor contributing to the phenotype in these children. However, the finding that five out of eight boys having the MAOA_LPR*2R presented learning difficulties and low working memory performance suggested that in some individuals, this genotype may have a major effect. On the contrary, the finding that three out of eight boys having normal or even high intelligence and school achievement indicates that having a MAOA_LPR*2R allele should not be considered as the sole cause of learning difficulties and should not be taken as a marker of school problems.
